# Immersive Virtual Reality–Based Cognitive Intervention for the Improvement of Cognitive Function, Depression, and Perceived Stress in Older Adults With Mild Cognitive Impairment and Mild Dementia: Pilot Pre-Post Study

**DOI:** 10.2196/32117

**Published:** 2022-02-21

**Authors:** KaiYan Zhu, QiongYao Zhang, BingWei He, MeiZhen Huang, Rong Lin, Hong Li

**Affiliations:** 1 The School of Nursing Fujian Medical University Fuzhou China; 2 Information Management Center Fujian Provincial Hospital Fuzhou China; 3 School of Mechanical Engineering and Automation Fuzhou University Fuzhou China; 4 Research Center for Nursing Theory and Practice Department of Nursing, Fujian Provincial Hospital Fuzhou China

**Keywords:** cognitive intervention, dementia, immersive virtual reality, mild cognitive impairment, cognitive impairment, virtual reality, VR, older patients, usability, memory, stress

## Abstract

**Background:**

The incidence of dementia is increasing annually, resulting in varying degrees of adverse effects for individuals, families, and society. With the continuous development of computer information technology, cognitive interventions are constantly evolving. The use of immersive virtual reality (IVR) as a cognitive intervention for older adults with mild cognitive impairment (MCI) and mild dementia (MD) is promising, although only few studies have focused on its use.

**Objective:**

The Chinese virtual supermarket (CVSM) IVR system was developed to provide a comprehensive and individual cognitive intervention program for older patients with MCI and MD. The aim of this study was to explore the feasibility and clinical effectiveness of this 5-week IVR-based cognitive intervention.

**Methods:**

A pretest-posttest study design was conducted with 31 older adults with MCI and MD from August 2020 to January 2021. All participants participated in a 5-week immersive virtual cognitive training program using the CVSM system. Feasibility was assessed as the incidence and severity of cybersickness symptoms and participant satisfaction based on questionnaires conducted after the intervention. Clinical effectiveness was evaluated using neuropsychological assessments, including several commonly used measures of cognitive function, depression, perceived stress, and activities of daily living. Measurements were obtained at baseline and after the intervention period.

**Results:**

A total of 18 patients with MCI (mean age 82.94 [SD 5.44] years; 12 females) and 13 patients with MD (mean age 85.7 [SD 4.67] years, 10 females) participated in this pilot study. Both groups showed significant improvements in all cognitive function measurements (*P*<.001). The MD group had a significantly greater improvement in general cognitive function compared to the MCI group in Montreal Cognitive Assessment Scale, Symbol Digit Modalities Test, Shape Trail Test, and Auditory Verbal Learning Test. Furthermore, an intervention effect was observed in the improvement of perceived stress (*P*=.048 for MD group, *P*=.03 for MCI group ).

**Conclusions:**

The use of the CVSM system may be effective in enhancing the cognitive function of patients with MCI and MD, including general cognitive function, memory, executive function, and attention. IVR technology enriches cognitive intervention approaches and provides acceptable, professional, personalized, and interesting cognitive training for older adults with cognitive impairment.

**Trial Registration:**

ClinicalTrials ChiCTR2100043753; https://trialsearch.who.int/Trial2.aspx?TrialID=ChiCTR2100043753

## Introduction

China is rapidly transforming into an aging nation. Based on the seventh national survey conducted by the National Bureau of Statistics, the number of older adults (aged ≥60 years) in China was 264.01 million (18.7% of the total population) at the end of 2020, including 190.63 million individuals aged ≥65 years (13.5% of the total population). It is predicted that there will be 400 million Chinese citizens aged ≥65 years by 2050, including 150 million aged ≥80 years [1]. Population aging is accompanied by an increased prevalence of mild cognitive impairment (MCI) and mild dementia (MD). In China, the prevalence of MCI in individuals ≥65 years is 10%-20%, and over 50% of these patients progress to dementia within 5 years. The incidence of dementia among individuals ≥60 years of age in China is predicted to increase from 14% in 2015 to 33% by 2050 [2,3]. The high prevalence of dementia in the older population is a significant social and economic burden on patients, patient families, and the existing health care system in China. The total cost associated with dementia is increasing at an alarming rate in China and is expected to exceed US $9.12 trillion by 2050 [4].

Although several studies regarding pharmacological treatments for MCI and MD have been conducted, the effectiveness of pharmacological treatments is limited. Therefore, safe treatment alternatives for MCI and MD have been developed, including cognitive interventions [5]. Cognitive training is among the most frequently used cognitive interventions and typically involves the repeated practice of a set of structured tasks [6,7]. In recent decades, many different cognitive training approaches aimed at improving and maintaining cognitive ability have been developed to reduce the progression of MCI and MD. However, most traditional cognitive training programs conducted by a neuropsychologist are based on face-to-face exercises [8,9], requiring the identification of a convenient meeting location, the coordination of schedules, and a dedicated training time. With the rapid development of computer science and technology, cognitive training exercises can now be delivered via computers or mobile technology, resulting in better patient compliance owing to the convenience of cognitive training [10-12]. Therefore, an increasing number of studies have focused on cognitive interventions based on computer technology.

Virtual reality (VR) is a new computer technology that was created with the development of multimedia technology and used in military science before being applied to the medical sciences [13]. VR is a computer-generated effect that can simulate a given scene through 3D graphics and other sensory experiences (vision, touch, and motion feeling) [14] by using special electronic devices such as computer keyboards, computer mice, speech/voice recognition, motion sensors, and haptic devices [15]. VR can be divided into 2 types according to the degree of immersion: nonimmersive and immersive virtual reality (IVR). The nonimmersive system is a desktop-based VR with low interaction (such as with a keyboard and joypad). IVR is characterized by the use of more interactive tools, including a head-mounted display or a cave automatic virtual environment, which allows patients to interact with such a virtual environment from the first-person perspective [16]. Nonpharmacological interventions based on IVR have been applied in clinical settings for pediatric patients, patients with psychotic disorders, older adults, and patients with MCI and MD [17-21]. Meta-analyses and systematic reviews have reported the effectiveness and advantages of VR for patients with MCI and MD. A recent systematic review reported that IVR has a potentially positive effect on global cognitive function, attention, and emotion [22]. Compared to traditional cognitive training, the VR environment is highly flexible and allows for cognitive training in environments that are either impossible or unsafe in real life. For example, IVR enables older adults with limited physical abilities to go shopping [23], ride a bicycle in a city [24], and cook [25] in a safe environment. In addition, the flexibility of VR allows for the adjustment of various parameters such as the duration of recall, types of stimuli [26], and numbers or similarities of distractors [27], ensuring that the settings match the patient’s individual capabilities.

Although IVR in the clinical setting has many advantages, the feasibility of its use among older adults remains controversial. A mixed method pilot study (n=10) examined the effects of a 15-minute interactive IVR forest experience on the level of engagement, apathy, and mood states of people with dementia and found that the use of IVR brought pleasure to participants but also increased their levels of fear and anxiety [28]. In addition, the extent to which IVR-based cognitive training can beneﬁt cognition in patients diagnosed with MCI or MD is unclear. Systematic reviews have also reported mixed results [29-31]. Therefore, more research regarding IVR-based cognitive training for patients with MCI and MD is needed. In this study, an IVR-based cognitive training program, the Chinese virtual supermarket (CVSM), was developed for use in older adults with MCI and MD, and a pretest-posttest study was conducted to evaluate the effects on neuropsychological outcomes in older adults with MCI and MD. We hypothesized that after the 5-week IVR-based cognitive training session, patients with MCI and MD would have significantly improved cognitive function and other health-related outcomes.

## Methods

### Recruitment

The proposed virtual cognitive assessment platform was installed for clinical testing at the Fujian Good Health Care Center. Older adults were recruited for the study via popular science lectures on VR, virtual game demonstrations, and VR experience activities. The participants were screened according to the inclusion and exclusion criteria. This study included 35 participants (21 with MCI and 14 with MD). All participants were ≥60 years of age, had normal or corrected-to-normal vision, and could communicate in Mandarin Chinese. Participants with MCI were diagnosed according to the Peterson diagnostic criteria [32]: clinically confirmed memory loss, intact or slightly impaired activities of daily living (score <23 for patients <75 years of age and <25 for patients >75 years of age), cognitive impairment based on the Chinese-Changsha version of the Montreal Cognitive Assessment Scale (MoCA) (abnormal value: MoCA score <13 for illiterate individuals, <19 for individuals with a primary school diploma, and <24 for individuals with a junior high school diploma or above), and preserved general cognitive function assessed using the Mini-Mental State Examination (MMSE) (24-30 points). Participants with MD were diagnosed by an experiential psychiatrist according to the International Classification of Diseases 10th revision [33] with a Clinical Dementia Rating score ≤1. Participants with severe audiovisual impairments (deafness, cataracts, or glaucoma), psychiatric and logic disorders (bipolar disorder, schizophrenia, stroke, Parkinson disease, or epilepsy), or a history of taking drugs affecting cognitive function in the 6 months prior to this study were excluded. All participants provided written informed consent for their participation in this study. This study was approved by the ethics committee of Fujian Provincial Hospital (K2020-06-006).

### Intervention Instruments

#### Virtual Environment

**Figure 1:**
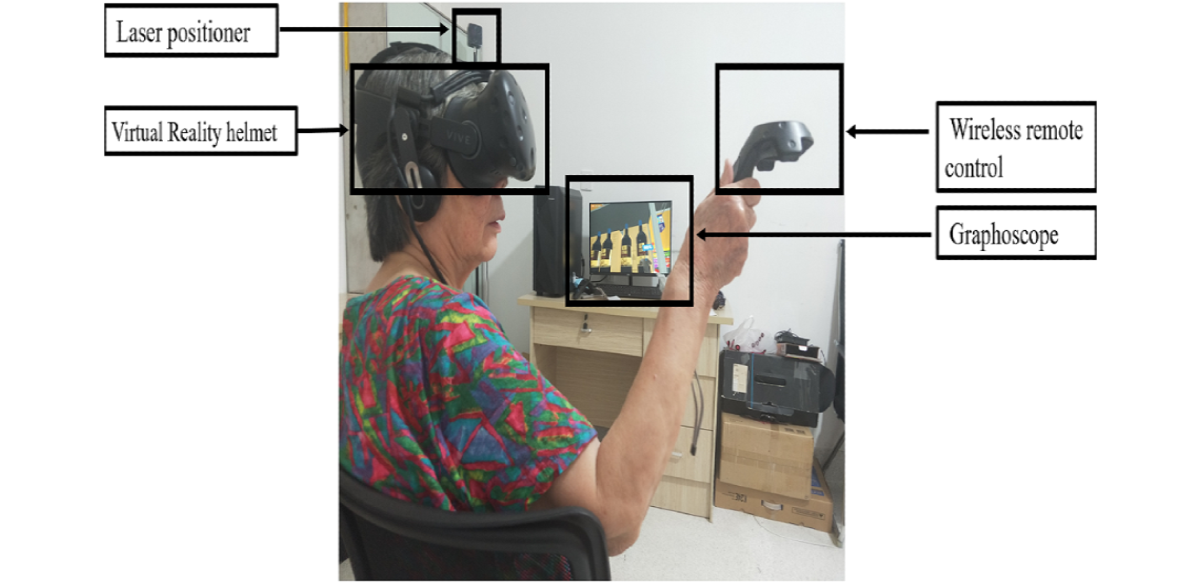
The Chinese virtual supermarket system.

The CVSM has applied for national computer software copyright, registration 2021SR0993516, which was developed by Fujian Provincial Hospital in collaboration with the School of Mechanical Design Manufacturing and Automation, Fuzhou University, and the Rhino Technology Group. The virtual environment of the CVSM was developed and rendered using a Unity 3D engine and was run on a Dell Precision T3600 PC with a CPU Intel I5-6400 processor and a GTX 1600 graphics card. The system required a capacity of 277 MB. The 3D virtual supermarket was presented using HTC VIVE Pro Eye, which provided a stereoscopic vision via 2 screens in front of the eyes (the resolution of the binocular combination was 2160×1200 pixels). The HTC Pro Eye allowed the participant to rotate his or her head for a 360° view of the virtual scene and to interact and walk freely in the virtual environment. With the help of an assistant, participants were asked to stand in an open room wearing a VR helmet (Figure 1). Through the headset, the participant viewed a rectangular virtual supermarket (9.8 m×18 m). Participants used a wireless remote control to select items within the virtual scene.

#### IVR-Based Cognitive Intervention

**Figure 2:**
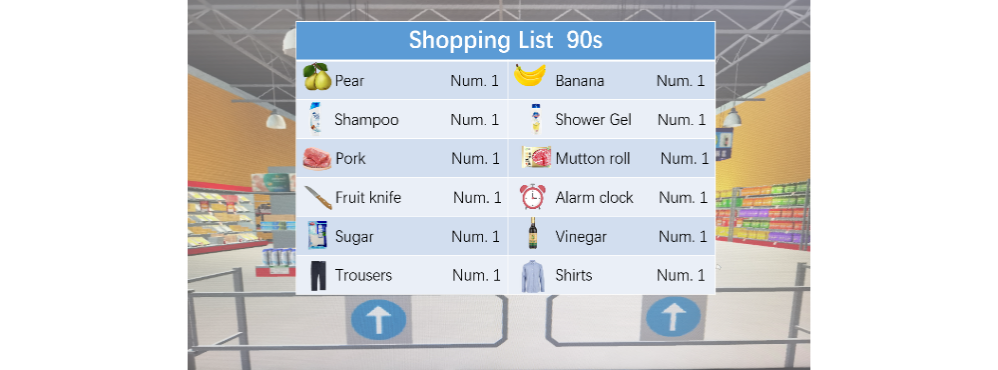
The shopping list of the Chinese virtual supermarket system.

**Figure 3:**
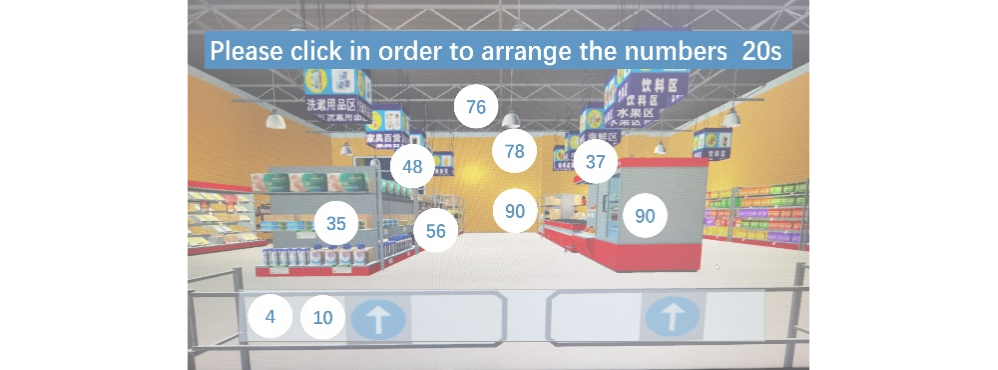
The number sorting game of the Chinese virtual supermarket system.

**Figure 4:**
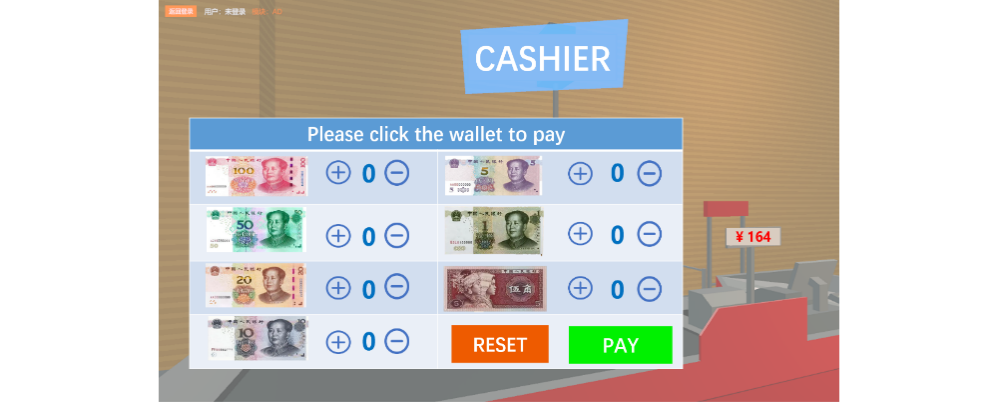
The payment interface of the Chinese virtual supermarket system.

The CVSM system was designed as a simple, easy-to-learn, personalized immersive VR cognitive management program for older adults and was divided into 3 modules: an operation learning module, a cognitive evaluation module, and a cognitive intervention module. In the operation learning module, participants were taught how to wear the 3D VR helmets and operate VR handles to interact with the virtual supermarket during 3 separate 10- to 15-minute sessions. Multiple exercises were used to help the participants adapt to the virtual environment. In the cognitive evaluation and cognitive intervention modules, the participants were presented with a list of 3-12 familiar virtual images and tags of common items (such as oranges or toothpaste) that they were asked to memorize within a minute and a half (Figure 2). After memorizing the list, the participants engaged in a number sorting game for 20 seconds (Figure 3) before entering the supermarket to buy the items on the memorized list. Then, participants were asked to locate the counter and pay in RMB bills, as shown in Figure 4. The final screen in the game confirmed that the payment was completed, and a report listing the quantities of products purchased, nonshopping list items, and the total time needed for completion was displayed. The participant’s task completion in the cognitive assessment module was set as the initial difficulty level in their cognitive training. The CVSM included 12 levels of difficulty according to memory quantity, delay time, calculation difficulty, and interval retrieval times. After completing 3 shopping sessions that included a maximum of 1 error each, the participant could increase his or her level of difficulty. Participants were instructed to train 3 times per week over a 5-week period and to complete 3 training sessions on each training day, with each session lasting 20-30 minutes. The entire cognitive training program ranges from simple to complex, involving training in multiple cognitive domains such as memory, attention, executive function, and calculation ability.

### Instruments to Measure Outcomes

A pretest-posttest study design was used to evaluate the effects of the VR-based cognitive intervention using the CVSM system in older adults with MCI and MD. Various measurements were obtained to evaluate feasibility and effectiveness of the IVR intervention. Demographic characteristics such as age, sex, and years of education were recorded during the initial evaluation. Trained psychologists assessed the participants.

#### Feasibility

Feasibility was assessed as the incidence and severity of cybersickness symptoms and participant satisfaction based on the answers provided in a questionnaire conducted after the intervention. The questionnaire was developed specifically for this study.

##### Severity of Cybersickness Symptoms

All participants were assessed for the severity of IVR by using the Simulator Sickness Questionnaire, which consists of 16 items regarding 3 factors: nausea (sweating, difficulty concentrating, or stomach awareness), oculomotor disturbance (headache, eyestrain, or blurred vision), and disorientation (head fullness, dizziness with eyes opened or closed, or vertigo). The severity of symptoms was categorized as none, mild, moderate, or severe. The Simulator Sickness Questionnaire score was calculated as the sum of the scores of the 16 items multiplied by 3.74, and it ranged from 0 to 179.52 [34].

##### Incidence of Cybersickness Symptoms

The incidence of cybersickness symptoms was calculated.

##### Satisfaction

The satisfaction questionnaire was used to assess the views and opinions of the participants regarding the intervention. Items were rated on a 4-point Likert scale (4=strongly agree, 3=agree, 2=neutral, and 1=poor). The total score ranged from 8 to 32, with higher scores indicating higher satisfaction. The causes of patient satisfaction were further investigated via open interviews.

#### Effectiveness

In this pilot study, the neuropsychological evaluation included several commonly used measures of cognitive function, depression, and perceived stress.

##### Cognitive Function

Global cognition was measured using the MMSE and MoCA, which have both been shown to have high test-retest reliability and be sensitive to changes in people with MD and MCI. MMSE and MoCA are respectively composed of 30 cognitive domain-related problems such as attention, language, word recall, time, and place positioning. The higher the score, the higher the cognitive level [35,36]. The effects of the treatments on memory function, executive function, and attention were assessed using the Auditory Verbal Learning Test (AVLT), the Shape Trail Test (STT), and the Symbol Digit Modalities Test (SDMT), respectively. In this study, the AVLT will be used to evaluate language learning and memory function, which includes 3 tests: immediate recall, short-term delayed recall, and long-term delayed recall [37]. The score of AVLT in this study is the sum of the 3 test scores. The higher the total recall score, the better the memory function. The STT was developed by Agnes Chan from the Chinese University of Hong Kong to evaluate executive function, and it consists of 2 components. The STT-A consists of 25 consecutive numbers—from 1 to 25. The STT-B was modified as 25 numbers enclosed in 13 circles (from 1 to 13) and 12 squares (from 1 to 12). Taking the sum of completion time as the scoring standard, the shorter the time, the better the executive function [38].The SDMT is used to assess the tester’s ability of attention, learning, conversion, and involves a substitution task using a coding key with 9 different abstract symbols, each paired with a numeral. Below the key, a series of these symbols was presented and the participant was asked to write down the corresponding number for each symbol. Participants need to complete as many of 115 items as they could in 90 seconds. The number of correct substitutions within this time was recorded as their score [39].

##### Depression

The Geriatric Depression Scale (GDS) is a self-report evaluation. The scale comprises 30 items: 10 items confirm depression if the answers are negative, while the remaining 20 items confirm depression if the answers are positive. Normal scores range from 0 to 10; a score of 11-20 indicates mild depression and 21-30 indicates moderate and severe depression [40].

##### Perceived Stress

The Chinese Perceived Stress Scale (PSS) is a scale for assessing individual stress. The scale mainly includes 14 self-assessment items: The 4th to 7th, 9th to 10th, and 13th items are used reverse score. The 1st to 3rd, 8th, 11th to 12th, and 14th items adopt positive scoring. The score of Chinese PSS equals total score minus 14. A higher score indicates that psychological pressure is higher, which is harmful to the physical and mental health [41].

### Statistical Analysis

Baseline data are presented using mean and standard deviation. Student *t* test and Wilcoxon signed rank test were used to analyze the results to evaluate the effect of the VR cognitive intervention in patients with MCI and MD. The *P* value was corrected by false discovery rate to reduce the risk of type I error. In order to determine the effective size of the results, the effect size calculator (Cohen *d*) for *t*test was used. The data were analyzed according to the intention-to-treat principle. Missing data were replaced with the individual's available data at baseline or were obtained by phone. All statistical analyses were conducted using the SPSS software (version 22.0; IBM Corp). Statistical significance was set at *P*<.05.

## Results

### Participant Characteristics

A total of 52 older adults showed interest in participating in this pilot study and were screened for eligibility; 17 participated in the upgrading and transformation of the CVSM system in the preparation stage and 35 agreed to participate in the feasibility study. Four participants (1 with MD and 3 with MCI) were unable to complete the study owing to physical limitations. Therefore, 18 participants with MCI (12 females and 6 males; mean age 82.94 [SD 6.44] years; mean years of education 11.0 [SD 3.97] years) and 13 participants with MD (10 females and 3 males; mean age 85.7 [SD 4.67] years; mean years of education 11.23 [SD 4.71] years) finally completed all courses of VR-based cognitive training as planned and were included in the final analysis. There were no significant differences between the MCI and MD groups at baseline with the exception of cognitive function (Table 1).

**Table 1:** Participants’ baseline characteristics (N=31).

Characteristic			MCI^a^ (n=18)		MD^b^ (n=13)		*P* value
**Sociodemographic data**							
	Age (years), mean (SD)	82.94 (6.44)		85.76 (4.67)		.19	
	Female, n (%)	12 (67)		10 (77)		.83	
	Education (years), mean (SD)	11.00 (3.97)		11.23 (4.71)		.88	
**Cognitive function, mean (SD)**							
	MoCA-CS^c^	21.56 (3.31)		13.92 (4.68)		<.001	
	MMSE^d^	26.06 (2.99)		18.54 (5.73)		<.001	
	AVLT^e^	38.11 (10.76)		25.38 (10.12)		.002	
	STT^f^	352.72 (132.79)		523.23 (307.71)		.04	
	SDMT^g^	21.83 (11.04)		12.85 (9.56)		.03	
**Psychosocial measures, mean (SD)**							
	PSS^h^	14.56 (12.05)		19.23 (15.28)		.35	
	GDS^i^	5.11 (4.23)		5.00 (4.63)		.95	

^a^MCI: mild cognitive impairment.

^b^MD: mild dementia.

^c^MoCA-CS: Chinese-Changsha version of the Montreal Cognitive Assessment Scale.

^d^MMSE: Mini-Mental State Examination.

^e^AVLT: Auditory Verbal Learning Test.

^f^STT: Shape Trail Test.

^g^SDMT: Symbol Digit Modalities Test.

^h^PSS: Perceived Stress Scale.

^i^GDS: Geriatric Depression Scale.

### Cybersickness Symptoms

**Figure 5:**
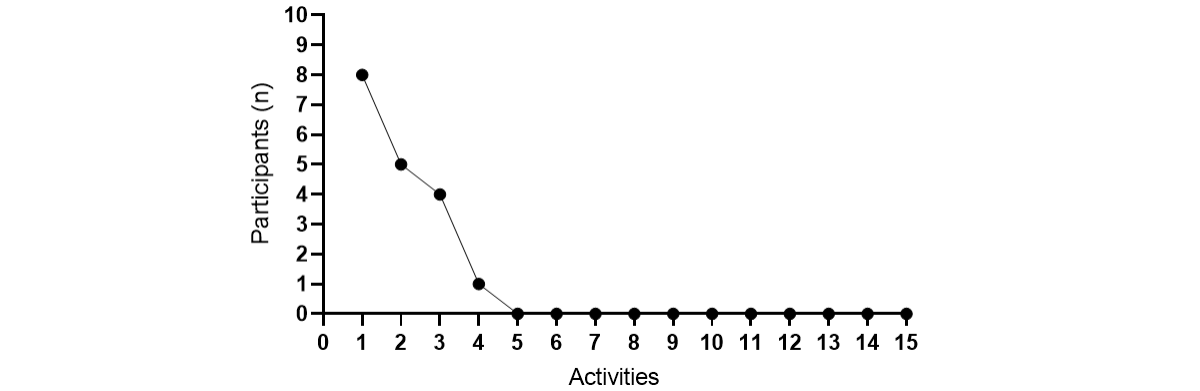
Frequency of cybersickness symptoms.

Eight participants had mild cybersickness symptoms in the first 4 activities (range of scores: 3.74-11.22 points). After 3 consecutive activities, the probability of the development of simulator disease was significantly reduced (Figure 5). After the fifth intervention, there were no reports of simulator disease. No adverse events such as injuries, falls, or quarrels were reported during the IVR interventions.

### Effectiveness of the Intervention

Statistical analysis was performed using the SPSS version 22.0. For comparison of the continuity variables of normal distribution, Student *t* test was used (Table 2). Otherwise, Wilcoxon signed rank test was used (Table 3). In order to reduce the risk of type I error, the *P* value was corrected by false discovery rate. As seen in Table 2 and Table 3, there is a significant difference between average pretest and posttest results in both groups (q<0.05), except GDS and PSS. In order to calculate the effect size of the results, Cohen *d* has been used. The effect size of the MD group results is significantly higher compared with that of the MCI group in MoCA, SDMT, STT, AVLT, and GDS.

**Table 2:** Statistical analysis of preintervention and postintervention results of mild cognitive impairment and mild dementia groups by using Student *t* test and effect size calculation results with Cohen *d*.

Group, index			Preintervention, mean (SD)		Postintervention, mean (SD)		*t* value *(df)*		*P* value		q value		Cohen *d*
**Mild dementia**													
	MoCA-CS^a^	13.92 (4.68)		21 (3.89)		–9.826 (12)		<.001		0.017		1.64	
	MMSE^b^	18.54 (5.74)		24.85 (5.74)		–7.697 (12)		<.001		0.017		1.09	
	SDMT^c^	12.85 (9.56)		17.31 (7.74)		–3.603 (12)		.004		0.009		0.51	
	PSS^d^	19.23 (15.28)		13.15 (10.95)		2.205 (12)		.048		0.056		0.45	
**Mild cognitive impairment**													
	MoCA-CS	21.56 (3.31)		25.67 (2.93)		–7.857 (17)		<.001		0.023		1.31	
	STT^e^	352.72 (132.79)		254 (66.18)		4.51 (17)		<.001		0.023		0.68	
	SDMT	21.83 (11.04)		27.22 (11.97)		–5.422 (17)		<.001		0.023		0.46	

^a^MoCA-CS: Chinese-Changsha version of the Montreal Cognitive Assessment Scale.

^b^MMSE: Mini-Mental State Examination.

^c^SDMT: Symbol Digit Modalities Test.

^d^PSS: Perceived Stress Scale.

^e^STT: Shape Trail Test.

**Table 3:** Statistical analysis of preintervention and postintervention results of mild cognitive impairment and mild dementia groups by using Wilcoxon test and effect size calculation results with Cohen *d*.

Group, index			Preintervention, mean (SD)		Preintervention, median (min-max)		Postintervention, mean (SD)		Postintervention, median (min-max)		*Z* value		*P* value		*q* value		Cohen *d*
**Mild dementia**																	
	AVLT^a^	25.38 (10.12)		24 (8-50)		38.53 (8.63)		38 (28-57)		2.97		.003		0.010		1.39	
	STT^b^	523.23 (307.71)		394 (227-1246)		338.76 (112.30)		306 (204-591)		–3.04		.002		0.014		0.79	
	GDS^c^	5.00 (4.63)		4 (0-16)		3.38 (3.52)		3 (0-12)		1.42		.15		0.154		0.39	
**Mild cognitive impairment**																	
	MMSE^d^	26.06 (2.99)		26.5 (16-29)		29.06 (0.72)		29 (28-30)		3.42		.001		0.003		1.37	
	AVLT	38.11 (10.76)		36 (14-64)		51.33 (8.28)		51.5 (32-68)		3.72		<.001		0.007		1.38	
	PSS^e^	14.56 (12.05)		8.5 (0-40)		9.11 (12.11)		3 (0-38)		2.18		.03		0.033		0.45	
	GDS	5.11 (5.24)		3.5 (0-20)		4.22 (6.31)		2 (0-24)		1.16		.25		0.245		0.15	

^a^AVLT: Auditory Verbal Learning Test.

^b^STT: Shape Trail Test.

^c^GDS: Geriatric Depression Scale.

^d^MMSE: Mini-Mental State Examination.

^e^PSS: Perceived Stress Scale.

### Satisfaction

The CVSM program received high user satisfaction ratings for all items (Table 4). All scores for cognitive intervention method/frequency, virtual environment, memory task, and interactive devices/modes were >3.50, indicating that the intervention was acceptable and positive. There were no complaints regarding the difficulty of the memory task. The visual and auditory supports (products, shopping lists, and hints) were reported to be clear and useful. The frequency of the intervention, comfort of the VR equipment, and difficulty of the operation received some neutral and poor ratings. One patient in the MCI group reported that the VR helmet was heavy, resulting in slight discomfort. Another patient in the MCI group reported that he did not like shopping in real life and was therefore neutral toward the VR supermarket scene. This patient also reported that the intervention was too frequent. Two patients in the MD group expressed concern about learning how to operate the IVR system, although they were able to complete the virtual shopping task with the guidance of the researchers.

**Table 4:** Participant satisfaction (N=31).

Items	Strongly agree, n (%)	Agree, n (%)	Neutral/poor, n (%)	Mean (SD)
Q1. Are you satisfied with the method of the IVR^a^ intervention?	21 (68)	10 (32)	0 (0)	3.68 (0.47)
Q2. Are you satisfied with the frequency of the intervention?	23 (74)	7 (23)	1 (3)	3.71 (0.52)
Q3. Did you enjoy doing the shopping?	18 (58)	12 (39)	1 (3)	3.55 (0.56)
Q4. Did you recognize all the products in VR^b^ supermarket?	31 (100)	0 (0)	0 (0)	4.00 (0)
Q5. Did you feel a sense of accomplishment after completing the shopping task?	17 (55)	14 (45)	0 (0)	3.55 (0.50)
Q6. Was it comfortable to wear the VR helmet?	25 (81)	5 (16)	1 (3)	3.77 (0.49)
Q7. Was it easy to learn VR handle operation?	26 (84)	3 (10)	2 (7)	3.74 (0.67)
Q8. Did you have a positive experience in using IVR?	25 (81)	6 (19)	0 (0)	3.81 (0.40)

^a^IVR: immersive virtual reality.

^b^VR: virtual reality.

## Discussion

### Principal Results

This study investigated the feasibility and effectiveness of a 5-week program for improving cognitive function in older adults with MCI and MD based on the increased use of IVR-based nonpharmacological interventions in the fields of cognitive impairment and geriatric preventive medicine. This is the first study to develop, implement, and evaluate the effects of an IVR-based cognitive training program in China. All participants who participated in the CVSM had improved executive function, attention, memory, and general cognitive function compared to baseline, which is consistent with the results of a meta-analysis of IVR-based cognitive training [42]. VR interventions are useful for patients with MCI or MD. Some studies [42,43] have reported limited positive effects in patients with MD and that the effects of VR were greater in patients with MCI than in patients with MD. However, in this study, the MD group had significantly greater improvement in general cognitive function than the MCI group. This difference in results may be due to the personalized difficulty setting of the CVSM system. An appropriate task difficulty setting and motivational incentives decrease the frustration of patients with MD when working on difficult tasks, enhancing the benefits of activated brain function [44]. Therefore, adjusting the appropriate task difficulty according to the basic cognitive level of patients with MD has a positive impact on improving the cognitive function of patients with MD.

The results of this study indicate that both groups had significantly improved perceived stress and depression after participating in the CVSM. VR has been observed to relieve anxiety and depression [45]. Some feasibility studies suggested that immersive virtual environment may promote the limited functioning of patients with cognitive impairment that affects communication, interaction, motivation, participation, and positive attitude toward others [46]. Therefore, the importance of virtual environment should be considered in cognitive training because the sense of existence in the virtual environment itself can enhance volitional motivation, allowing people to constantly deal with external stimuli and adapt to the changing environment in cognition [47]. There were few reports of mild dizziness during the early stages of the cognitive training period in this study, and simulator-related symptoms decreased as the number of cognitive training sessions increased. These results suggest that the CVSM is a feasible IVR system for older adults with MCI and MD. The low incidence of simulator disease in this study may be associated with the frequency and time of cognitive training programs. The neural mismatch model [48] indicates that unpleasant symptoms occur when the sensory information is inconsistent with the individual’s past experiences. Therefore, older adults are prone to discomfort when they enter some VR environments. The use of adaptation training programs that last for several days and the gradual increase of time spent using the simulator during a single training session can help prevent the discomfort associated with the simulator [49].

Some intervention studies have reported high dropout rates of older adults in cognitive training based on technical support [43,50]. Cognitive training based on Immersive Virtual can be regarded as an effective method to enhance user participation, which contributes to the positive results of intervention [51]. However, older adults are less exposed to modern digital technology in their daily life. Therefore, not all can understand and accept it. In order to overcome this limitation, this study carried out many interesting lectures related to VR and experience activities in nursing homes during the recruitment of research objects, so as to increase older adults' understanding of virtual technology and shorten the “digital gap” between older adults and technology. The results of the satisfaction survey suggest that the participants in this study were highly engaged during the intervention and had a positive attitude toward IVR-based cognitive training. The majority of the participants were attracted to this novel cognitive training method.

### Limitations

This study is not without limitations. First, our pretest and posttests were identical. Older adults received a total score for each pretest but were not notified which answers they got correct on the pretest. Nevertheless, if they remembered the questions in the pretest, the identical pretests and posttests may have exaggerated the posttest scores. Second, the sample size was small and the study did not include a control group and a long-term follow-up analysis. However, according to the Virtual Reality Clinical Outcomes Research Experts framework [52], researchers are required to conduct early testing with a focus on feasibility, acceptability, tolerability, and initial clinical effectiveness before a standardized randomized controlled trial can be started. Therefore, this study is beneficial as it reduces the risk of conducting a randomized controlled trial with an intervention that has not undergone thorough testing. The effects and mechanisms of IVR-based cognitive training on the cognitive performance of patients with MCI and MD should be studied using high-quality randomized controlled trials.

### Conclusion

In conclusion, this pilot study validated the feasibility and effectiveness of IVR-based cognitive training by using a novel CVSM system in older adults with MCI and MD. The results of this study support the use of IVR-based cognitive training in this patient population.

## Abbreviations

AVLT: Auditory Verbal Learning Test
CVSM: Chinese virtual supermarket
GDS: Geriatric Depression Scale
IVR: immersive virtual reality
MCI: mild cognitive impairment
MD: mild dementia
MoCA: Montreal Cognitive Assessment Scale
MMSE: Mini-Mental State Examination
PSS: Perceived Stress Scale
SDMT: Symbol Digit Modalities Test
STT: Shape Trail Test
VR: virtual reality
